# Presumed Protective Role for Anti-Hepatitis B Virus Antibodies Against COVID-19 Severe Cases: A Clinical Study Confirming *in silico* Hypothesis

**DOI:** 10.3389/fmed.2022.909660

**Published:** 2022-07-08

**Authors:** Mariem Gdoura, Raoua Touati, Sana Kalthoum, Rania Ben Slama, Nouel Fatnassi, Mehdi Mrad, Lamia Ammari, Nozha Brahmi, Amira Ben Jazia, Nahed Hogga, Henda Triki, Sondes Haddad-Boubaker

**Affiliations:** ^1^Laboratory of Clinical Virology, WHO Regional Reference Laboratory for Poliomyelitis and Measles for the EMR, Institut Pasteur de Tunis, University of Tunis El Manar, Tunis, Tunisia; ^2^LR20IPT10 Laboratory of Virus, Host and Vectors, Institut Pasteur de Tunis, University of Tunis El Manar, Tunis, Tunisia; ^3^Department of Clinical Biology, Faculty of Pharmacy of Monastir, University of Monastir, Monastir, Tunisia; ^4^Centre National de Veille Zoosanitaire, Tunis, Tunisia; ^5^Laboratory of Biochemistry, Institut Pasteur de Tunis, University of Tunis El Manar, Tunis, Tunisia; ^6^Infectious Diseases Departement, Rabta Hospital, Tunis, Tunisia; ^7^Intensive Care Service, Emergence Medical Assistance Center, Tunis, Tunisia

**Keywords:** COVID-19, correlation, antibody titer, Hepatitis B, SARS-CoV-2

## Abstract

**Background:**

Severe acute respiratory syndrome coronavirus 2 (SARS-CoV-2) is responsible for COVID-19 disease which is known to have a broad clinical spectrum, from asymptomatic to critical presentation leading to death. Many researchers have investigated the factors impacting the course of the disease. Our previous *in silico* study suggested a possible protective effect of Hepatitis B, Tetanus and Measles vaccines against COVID-19. In continuity, we conducted a cross-sectional clinical study in order to confirm our *in silico* assumptions regarding the HBs-Ag antibodies.

**Methods:**

A representative sex- and age-matched sample of patients with confirmed COVID-19 was selected (*n* = 340). All clinical presentations were equally represented. Using an ELISA test, each patient benefited of a serology for the detection and measurement of the anti-HBs specific IgG antibodies. The obtained results allowed determining the different correlations between these antibody titers and the disease severity. The R^®^ software and the MedCalc^®^ software served to calculate the Spearman's coefficient of rank correlation (rho) for the obtained titers per severity group as well as the different other calculations and figure representations.

**Results:**

A significant positive correlation was found with the anti-HBs titers (rho = 0.107; *p* = 0.04). High anti-HBs titers were significantly associated with the mild presentation of COVID-19. A significant difference was found between the obtained titers per severity class (chi-2 test, *p* = 0.03).

**Discussion/Conclusion:**

Our findings demonstrated that anti-HBs titers were significantly higher for patients having mild COVID-19 presentations. We presume that being immunized against the HB may play a protective role in the course of the disease. Our study provided more key elements in understanding the disparity of the clinical spectrum among regions.

## Introduction

The emergence and the spread of the severe acute respiratory syndrome coronavirus 2 (SARS-CoV-2), responsible for COVID-19, have caused global public health, economic, and social crises (1, 2). While most cases of COVID-19 are mild to moderate, a significant fraction may develop severe to critical clinical presentations leading to death (3). As of 29 March 2022, 6,127,981 COVID-19-related deaths, worldwide, have been reported to the WHO (4). An uneven mortality distribution across the WHO regions is noteworthy. This unequal distribution is even deeper between countries throughout the regions as they are reporting very different case fatality ratios (5). As an example, within the region of the Americas, Venezuela reported only 19 deaths per 100,000 inhabitants, whereas the rates reported by Peru exceeded 600 per 100,000 inhabitants (4). Thomas et al. (6) have gone even further by examining 19 different cities in the United States and concluded to non-uniform COVID-19 severity within the same city level. Presently, almost 2 years after the virus emergence, we are still learning about the risk factors of severe COVID-19 outcomes (7). Demographic risk factors such as advanced age as well as underlying medical conditions have evident impact on the COVID-19 severity (8). In addition, many other assumptions have been made in order to explain these substantial striking disparities such as human leukocyte antigen (HLA) polymorphism, race and ethnicity, environmental conditions, nutrition, and microbiome (9–12). The most plausible hypotheses were those involving the immune response mechanisms (13, 14), and it was suggested that some vaccines could enhance the innate immune response (15, 16). Indeed, even before the emergence of the COVID-19 pandemic, it was described that the large-scale use of the BCG vaccine (Bacillus Calmette-Guérin) has beneficial nonspecific effects on the immune system by protecting against other infectious diseases and reducing the non-tuberculosis child mortality (17). Recently, Curtis et al. (18) suggested that it might be involved in the protection against COVID-19 as it could reduce viraemia and thus enhance rapid recovery and less severity. Anbarasu et al. (19) also suggested that the extensive pediatric vaccination with MMR vaccines (Measles, Mumps, and Rubella vaccines) has induced interferon (IFN) secretion and activated natural killer cells, offering, thereby, natural immunity against SARS-CoV-2 in the young population.

**Figure 1:**
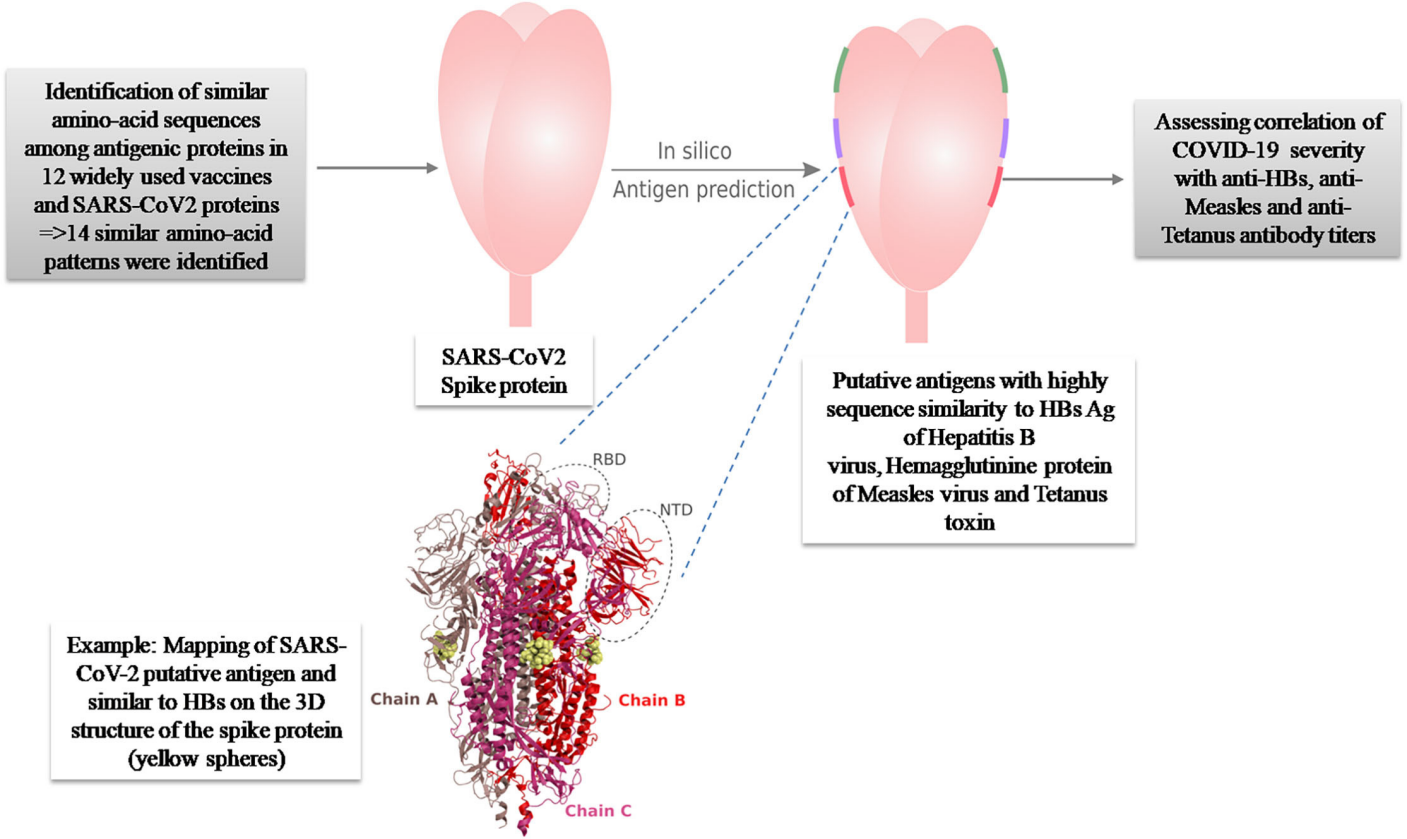
Summary of the original *in silico* hypothesis suggesting an antigenic potential of three amino-acid patterns present in HBs Ag of Hepatitis B, Hemagglutinine protein of Measles virus and Tetanus toxin, against SARS-CoV-2 (20).

In a previous paper, we investigated the putative protective role against COVID-19 of 12 widely used vaccines, including live attenuated (BCG, Oral Polio Vaccine, MMR vaccines) and inactivated ones [tetanus, *Corynebacterium diphtheriae, Bordetella pertussis*, Hepatitis B (HB), Hepatitis A, *Haemophilus influenzae* type B (Hib), and *Streptococcus pneumoniae* vaccines (PCV10)] (20). A total of 30 antigenic proteins were investigated. Using a package of the *in silico* analysis tools, we performed amino acid sequence alignments and hot spot analysis. Among the investigated antigenic proteins, 14 proteins presented similar amino acid patterns in eight different vaccines. Structural and antigenicity tests (B-cell and T-cell epitope predictions) identified three segments in Hepatitis B, Measles and Tetanus proteins presented antigenic properties that can induce putative protective effect against SARS-CoV2 (Figure 1).

Hepatitis B is a vaccine-preventable disease; however, it is the world's most common liver infection. The WHO estimates that 296 million people were living with chronic HB infection in 2019, with 1.5 million new infections each year (21). National and international efforts are being made in order to improve the vaccine coverage through systematic childhood and health workers vaccination. We were wondering whether this unequal immunization between countries may justify the COVID-19 severity variability. Within this vision, and in continuity to the previous *in silico* study (20), we investigate in this work the putative protective role of the anti-HBs-Ag specific IgG of the HBV against SARS-CoV-2 using real samples from patients who recovered from COVID-19. Our aim was to identify the statistical correlation between the corresponding antibody titers and the disease severity among a large sample size.

## Methods

### Ethics Statement

This study was performed under ethical standards according to the 1964 Declaration of Helsinki and its later amendments. The samples were collected in the context of COVID-19 diagnostic activities. They were used in this study after de-identification with respect to patient anonymity and after the approval of the Bio-Medical Ethics Committee of Pasteur Institute of Tunis, Tunisia (2020/14/I/LR16IPT/V1).

### Study Population

This cross-sectional study was conducted between May and June, 2021, in the Laboratory of Clinical Virology (LCV) of Pasteur Institute of Tunis. The included sera were randomly selected from the LCV bio-bank; they were collected during the pandemic from patients with different clinical presentations and then carefully stored in a −40°C freezer in accordance with the BAOBAB^®^ LIMS storing application (22). A total of 340 patients with COVID-19 infection were enrolled, matched for age and sex, and classified into “asymptomatic,” “mild,” “moderate,” and “severe” according to the United Stated National Institutes of Health (NIH) definitions update of 19 October 2021 (3). Each group contained 85 patients. All selected patients were not vaccinated against SARS-CoV-2 and the infection with COVID-19 was confirmed either by real-time reverse transcription PCR (RT-qPCR) assessed on nasopharyngeal swab by a WHO-approved in-house protocol (the Hong Kong University, China, RT-qPCR protocol) (23) or by SARS-CoV-2-specific antibody seroconversion detected by the commercial test Elecsys^®^ Roche^®^ total anti-nucleoprotein antibodies.

### Clinical Data Collection

The socio-demographic data of patients and information on clinical features, co-morbidities, and exposure or contact history with COVID-19 patients were collected.

### Detection and Measurement of the Anti-HBs

Detection and measurement of the anti-HBs IgG-specific antibodies were carried out by the commercial *in vitro* diagnostics (IVD)-validated immunoassay: an anti-HBs enzyme immunoassay kit, ETI-AB-AUK-3, manufactured by the DiaSorin^®^ S.p.A., Italy (REF: P001603). This assay is based on a direct and non-competitive sandwich ELISA. It enables detection of anti-HBs IgG-specific antibodies using wells coated with heat-treated human HBs-Ag. The measurement of the anti-HBs specific antibodies depends on the use of standardized calibrators that were referenced against WHO anti-Hepatitis B Immunoglobulin 1st International Reference Preparation, 1977. This kit is recommended for measuring the anti-HBs titers whether acquired as a result of natural HBV infection or after vaccination. The sensitivity and specificity of this kit are 99.11% CI 95% (98.18–99.64%) and 98.21% CI 95% (97.07–99.00%), respectively, following the manufacturer's instructions. The testing procedures and result interpretation were conducted according to the kit instructions: a titer lower than 10 mIU/mL indicates that the patient is not immunized against the HBV; a titer higher than 10 mIU/mL is correlated to an efficient immunity against the virus; however, a titer more than 100 mIU/mL is recommended for the vulnerable populations such as the health care workers. This classification also aligns with the WHO recommendations (24).

### Statistical Analysis

To explore the relationship between anti-HBs specific antibody titers and the severity of COVID-19 cases, the sample size was first calculated with 80% power of the test, an alpha value set to 0.05, and a correlation value of 0.3 using Epitools^®^ (25). The sample size was calculated using the Sample Size Calculators website (26). The Spearman's rank correlation coefficient was used to determine the correlation between anti-HBs specific antibody levels and the SARS-CoV-2 severity of cases. Continuous data were presented in median and ranges and categorical data were presented in numbers and percentages. All statistical analyses were performed using the MedCalc^®^ Software (version 18.2.1) and the R^®^ software (version 3.4). A *p*-value of <0.05 was considered statistically significant.

## Results

### Demographic Features of the Tested Population

**Figure 2:**
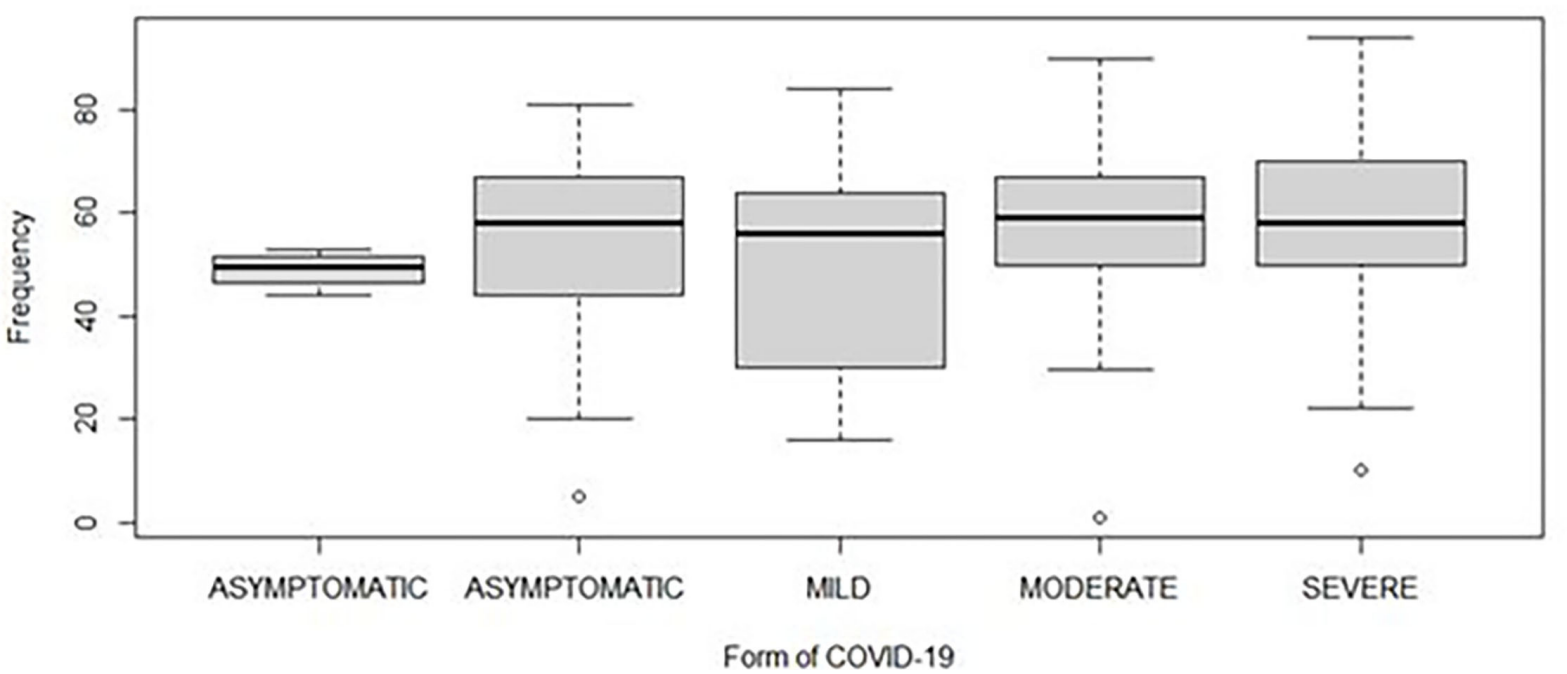
Distribution of the age of patients according to different severities of COVID-19. The horizontal line inside each box represents the median value of the age with interquartile ranges. The dots represent patients.

This study included 340 patients that were stratified according to COVID-19 disease severity into four groups: “asymptomatic,” “mild,” “moderate,” and “severe.” The required sample size was 85 patients in each group. Among the 340 patients, 160 were female (47%) and 180 (53%) were male. The sex ratio male/female (M/F) was estimated at 1.1. The mean age for all groups was 54 years (1–94 range; Figure 2).

### Serological Results

**Figure 3:**
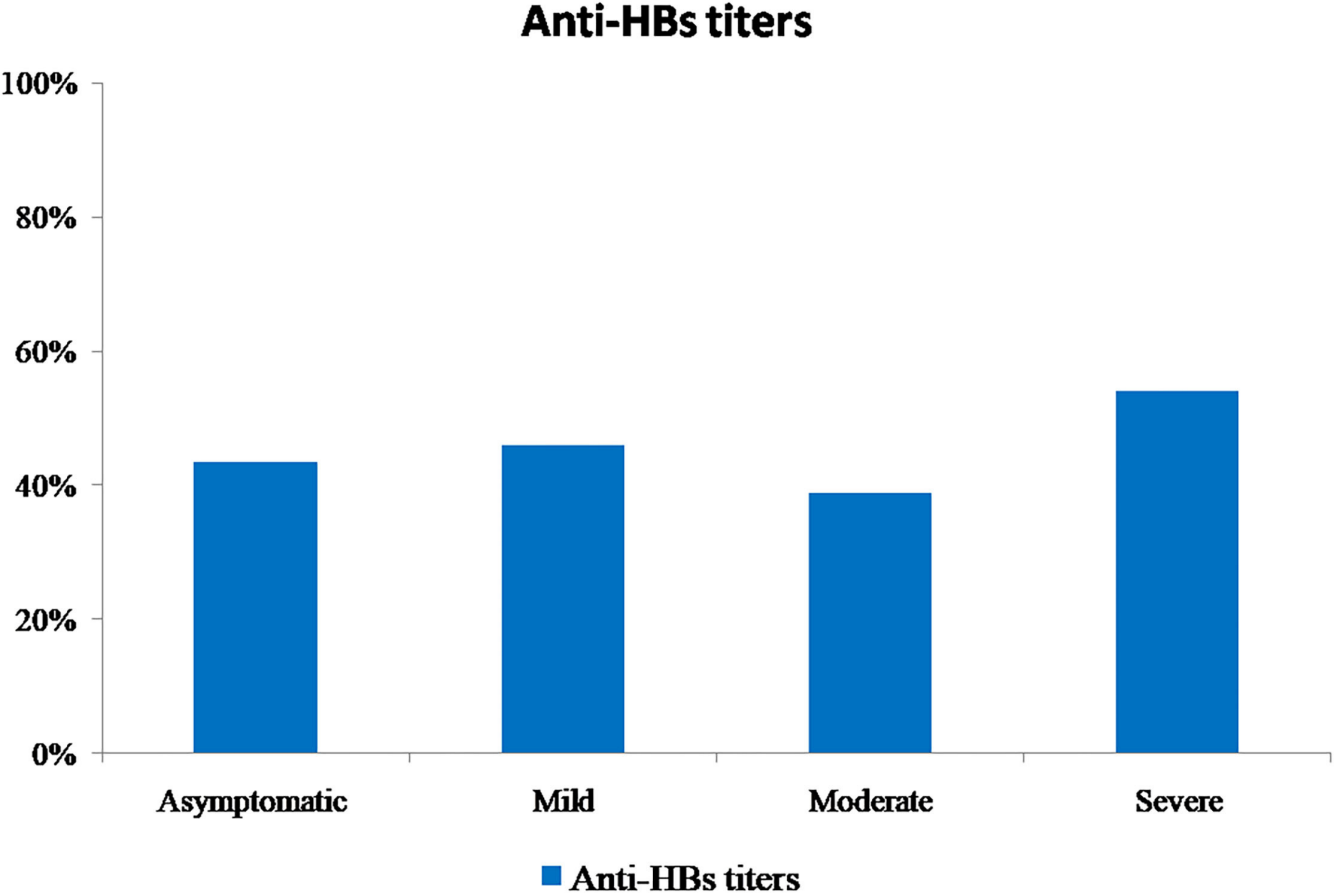
Proportions of Hepatitis B sero-positive patients per COVID-19 severity classes.

All sera were tested for the presence of anti-HBs antibodies. In total, 54.4% (*n* = 185) of patients did not have detectable anti-HBs antibodies. For the remaining proportion of patients who had positive anti-HBs antibodies (*n* = 155, 45.6%), it was distributed according to the COVID-19 disease severity group as shown in Figure 3. Regarding the antibody measurement, the titers ranged from 14 to 2,390 mIU/mL, with a median of 100 mIU/mL. For the “asymptomatic” class, titers ranged from 15 to 2,380 mIU/mL; for the “mild” class, titers ranged from 15 to 2,390 mIU/mL; for the “moderate” class, titers ranged from 15 to 2,220 mIU/mL; and for the “severe” class, titers ranged from 14 to 2,265 mIU/mL. A significant difference was found between the obtained titers per severity class (chi-2 test, *p* = 0.03). In addition, a significant reverse correlation was found between the patients' ages and the obtained anti-HBs antibody titers (rho = −0.176; *p* < 0.05); this association demonstrated that higher anti-HBs titers were detected in children and the adult patients than that in older age.

### Correlation Analysis

**Figure 4:**
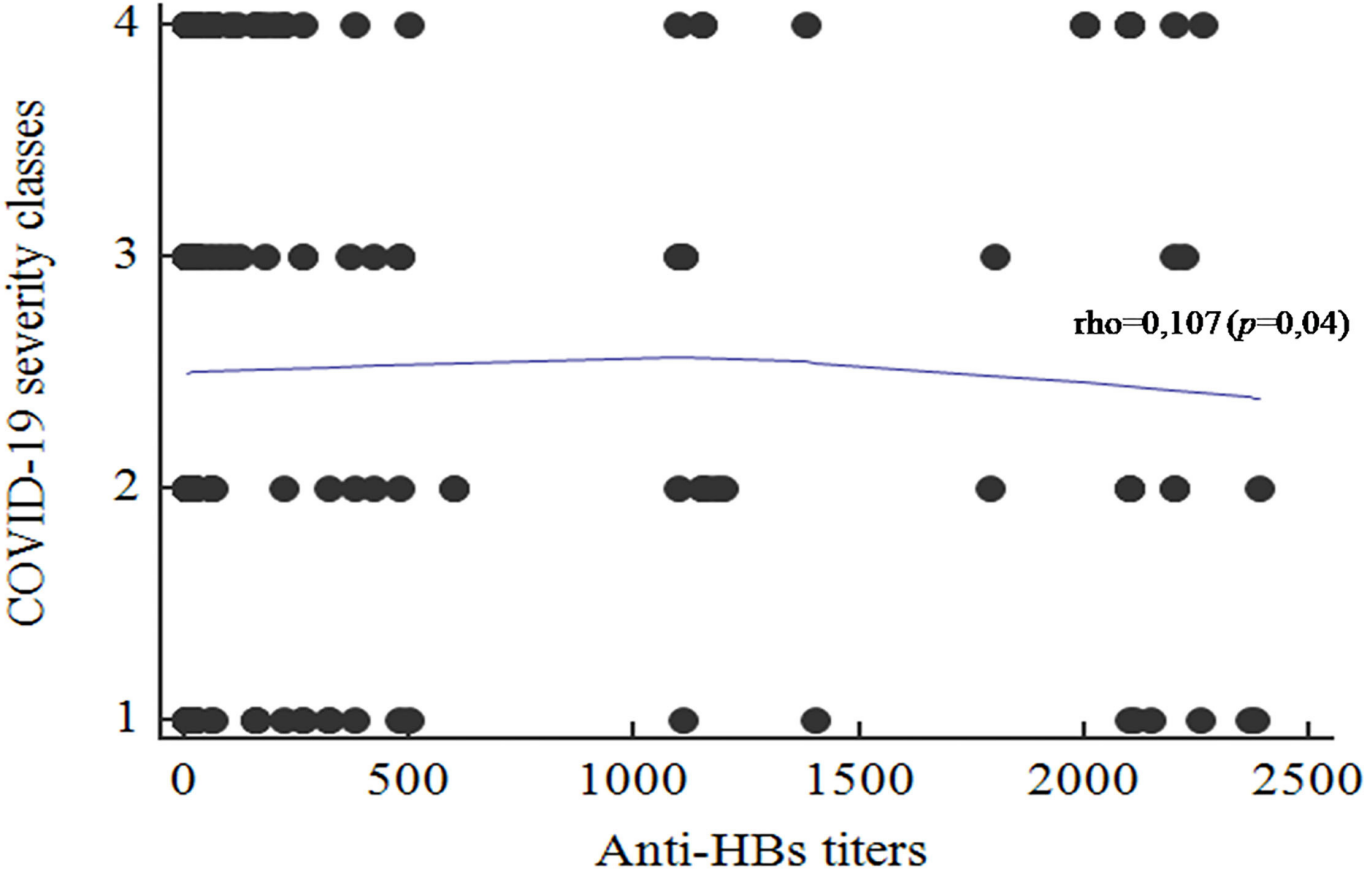
Correlation between the anti-HBs titers and the COVID-19 disease severity classes (1, Asymptomatic; 2, Mild; 3, Moderate; 4, Severe.

Taking into consideration the different obtained titers, the correlation with the COVID-19 severity classes was performed. A significant positive correlation was found between the anti-HBs titers and the COVID-19 severity classes (rho = 0.107; *p* = 0.04; Figure 4). High anti-HBs titers were significantly associated with mild presentation of COVID-19. In contrast, patients with severe clinical presentations had lower antibody titers.

## Discussion

In this article, we investigated the potential role of a routinely used vaccine against HBV in preventing severe clinical presentations of the COVID-19 disease. Our aim was to confirm our previous *in silico* findings. Indeed, we identified similar amino acid pattern in HBs-Ag protein of HBV and SARS-CoV-2 with antigenic properties (20) (Figure 1). We have attempted to identify whether there is a correlation between HBV antibody titers and the severity of SARS-CoV-2 infection, using sera of patients who recovered from COVID-19.

Several studies have previously suggested a potential cross protection of post-vaccine antibodies toward severe to critical outcomes of the SARS-CoV-2 infection (17–19, 27, 28) and, as the experimental identification of the incriminated epitopes is challenging, many studies had recourse to *in silico* approach. Indeed, bioinformatic and computational biology provides a valuable contribution in the current COVID-19 emergency context, thanks to its rapidity and low cost in comparison to wet-laboratory and clinical investigations (29). However, *in vitro* investigations remain a more solid way to support conclusions. Thus, a total of 340 patients with confirmed SARS-CoV-2 infection were enrolled in this study, matched for age and sex, and classified into different clinical presentations (*n* = 85/group) according to the National Institute of Health definitions (3). To our knowledge, our study investigated the largest population for the determination of vaccine correlation as compared to previous ones and reported, for the first time, that the presence of high anti-HBs titers in patients' sera may be associated with a significant protective role against the COVID-19 disease. Gold et al. investigated only a total of 80 patients with COVID-19 for the relationship with MMR vaccine (30). Al Balakosy et al. also determined the anti-Measles IgG antibodies on a total of only 78 patients with COVID-19 (31).

The active substance in the HB vaccine is the viral surface protein HBs-Ag, obtained by the yeast-derived recombinant vaccine biotechnology, in most commercially available vaccines (32). The immunogenic fraction is the amino acid hydrophilic region, referred as the common *a* determinant present in the HBs-Ag (subtype HBs-Ag-adw2 or HBs-Ag-adr) (32, 33). These two antigenic epitopes were investigated in our previous *in silico* study and we showed that the amino-acid sequence PGTNTSN in the Spike protein of SARS-CoV-2 (position 600–606) matches with the predicted epitope TNTSN in the HBs-Ag-adr (20). The pattern PGTNTSN corresponded to an exposed site in the S protein with a high accessible surface area value and presented probing spheres mimicking the CDR antibodies, that was in line with a potential implication in the B-cell mediated response. Furthermore, it was also described by Tajiri et al. (34) that two regions of HBsAg (residues 104–123 and 108–123), containing the epitope matching the PGTNTSN segment of SARS-CoV-2, were able to bind with two human monoclonal antibodies. This highlighted the immunogenic ability of these segments. Indeed, high-antibody titers were found among the studied population within the mildly infected group, with a significant positive correlation (rho = 0.107; *p* = 0.04) which is in concordance with our previous *in silico* findings (20). Therefore, we conclude that epitopes in regions of HBsAg (residues 104–123 and 108–123) matching the PGTNTSN segment of RBD may induce a cross protection against SARS-CoV-2. *In vivo*, this might lead either to a direct inhibition of the early cell-entry phases or to an indirect non-specific steric clutter. Nevertheless, only experiments such as sero-neutralization assay may support such findings.

Relying on clinical observations, Chen et al. (35) and Wu et al. (36) reported that SARS-CoV-2 infection in patients infected with HBV could facilitate the development of liver injury which is associated with adverse outcomes. Accordingly, it can also be suggested that HBV vaccination may also indirectly protect patients from these adverse outcomes (37). Furthermore, the possible protective role of HBV vaccine against other diseases, such as lymphoma, was reported by two previous studies (38, 39). Presently, the safe and efficient HB vaccination is highly recommended for all children worldwide. However, the WHO reports widely variable coverage rates from country to country, depending on the respective national strategies (24). From another point of view, it is well known, today, that after an initial vaccination, anti-HBs antibody titers may decline over years (40), making elderly less immunized than the other age groups. So, taking into consideration that the severe COVID-19 presentations are more frequent among elderly (8), we attempt to speculate that the loss of anti-HBs antibodies may be in line with our hypothesis. In our study, we performed a serum measurement of the anti-HBs antibodies regardless of the vaccination history because only the presence of anti-HBs at a protective level may indicate an effective protection whatever it was obtained through vaccination or natural infection. Furthermore, the non-response rates to the HBV vaccine range from 5 to 10% in total population and can reach 40% among patients with diabetes and kidney failure (40). For this study, the patients came from Tunisia which is a low-endemic country as result to the introduction of vaccination since 1995. Indeed, on the basis of the most recent national household-based cross sectional and serological survey in 2015, the national point prevalence of HBs-Ag was 1.7% [95% CI (1.6–1.9%)] and the vaccine effectiveness was 88.6% [95% CI (81.5–93.0%)] (41).

Epitopic similarity between different virus' antigens is a well-described phenomenon that has various implications on pathogenicity comprehension, diagnosis methods, and even treatment opportunities. It has been demonstrated during the emergence of Zika virus, for example, that there is a strong structural similarity with the matrix and the envelope antigens of dengue and West Nile viruses, i.e., within the same family of Flaviviridae (41). It has been reported, recently, that these antigenic cross-reactivities have impeded the IgM-specific antibody serology assays (42). Furthermore, beyond the viruses, it was demonstrated that there is a molecular mimicry between self-human and viral antigens, which might trigger autoimmune diseases in genetically predisposed individuals (43). For instances, viruses such as Coxsackievirus, Mumps virus, Rubella virus, and Hepatitis C virus were incriminated in inducing the type 2 diabetes, again by exhibiting molecular mimicry with the host proteins (44). For the SARS-CoV-2, the previously reported epitopic similarity (20) and the current findings may suggest a possible cross protection. Nevertheless, it should be supported by larger statistic investigation and further lab experiments such as sero-neutralization assays. Our findings may be unique and encourage other studies targeting previously used antiviral to be tested against SARS-CoV-2 infection, especially those used against HBV chronic infection. Indeed, García-Trejo et al. published an argument repurposing the Lamivudine (nucleotide/nucleoside analogs); they provided *in silico* docking evidence suggesting that Lamivudine may bind and possibly work as an inhibitor of the SARS-CoV-2 RdRp RNA polymerase (45). The telbivudine also was suggested as a fighting drug for COVID-19 (46). These data underline the possible similarity with gene sequences between HBV and SARS-CoV-2.

## Conclusion

In the COVID-19 crisis context, clinical research is escalating and providing mounting evidence that immunity background plays a crucial role in deciding the course of the disease. Our findings have placed emphasis on HBV, linking anti-HBs high sero-positivity to the COVID-19 minor severity, as its antigenic properties were consistent with its putative neutralizing capacity. Although findings were significant, larger population investigation may further support the obtained correlation. Also, it will be interesting to investigate the sero-neutralization effects of anti-HBs antibodies using different SARS-CoV-2 variants of interest.

The observed associations between anti-HBs antibody titers and the COVID-19 disease course may explain the geographical disparity worldwide of the COVID-19 severity, along with all the suggested risk factors. We believe that it is still important to dig into the protective and risk factors that have led to the large number of deaths inherent to COVID-19 especially in the context of SARS-CoV-2 variant emergence.

## Data Availability Statement

The original contributions presented in the study are included in the article/supplementary material, further inquiries can be directed to the corresponding authors.

## Ethics Statement

The studies involving human participants were reviewed and approved by Bio-Medical Ethics Committee of Pasteur Institute of Tunis, Tunisia. Written informed consent to participate in this study was provided by the participants or their legal guardian/next of kin.

## Author Contributions

MG, SH-B, and SK: conceptualization, methodology, and original draft preparation. RT, RS, and NH: investigation. RT, SK, and NF: data curation. MM, LA, NB, and AJ: sample and data collection. MG, SH-B, SK, and HT: writing—review and editing, supervision, and project administration. All authors contributed to the article and approved the submitted version.

## Funding

This study was funded by the Tunisian Ministry of Higher Education and Scientific Research (LR20IPT10).

## Conflict of Interest

The authors declare that the research was conducted in the absence of any commercial or financial relationships that could be construed as a potential conflict of interest.

## Publisher's Note

All claims expressed in this article are solely those of the authors and do not necessarily represent those of their affiliated organizations, or those of the publisher, the editors and the reviewers. Any product that may be evaluated in this article, or claim that may be made by its manufacturer, is not guaranteed or endorsed by the publisher.
